# Functional Desaturase *Fads1* (*Δ5*) and *Fads2* (*Δ6*) Orthologues Evolved before the Origin of Jawed Vertebrates

**DOI:** 10.1371/journal.pone.0031950

**Published:** 2012-02-22

**Authors:** Luís Filipe Costa Castro, Óscar Monroig, Michael J. Leaver, Jonathan Wilson, Isabel Cunha, Douglas R. Tocher

**Affiliations:** 1 CIIMAR – Interdisciplinary Centre of Marine and Environmental Research, CIMAR Associate Laboratory, University of Porto, Porto, Portugal; 2 Instituto de Acuicultura Torre de la Sal (IATS-CSIC), Castellón, Spain; 3 Institute of Aquaculture, School of Natural Sciences, University of Stirling, Stirling, Scotland, United Kingdom; Laboratoire Arago, France

## Abstract

Long-chain polyunsaturated fatty acids (LC-PUFAs) such as arachidonic (ARA), eicosapentaenoic (EPA) and docosahexaenoic (DHA) acids are essential components of biomembranes, particularly in neural tissues. Endogenous synthesis of ARA, EPA and DHA occurs from precursor dietary essential fatty acids such as linoleic and α-linolenic acid through elongation and *Δ5* and *Δ6* desaturations. With respect to desaturation activities some noteworthy differences have been noted in vertebrate classes. In mammals, the *Δ5* activity is allocated to the *Fads1* gene, while *Fads2* is a *Δ6* desaturase. In contrast, teleosts show distinct combinations of desaturase activities (e.g. bifunctional or separate *Δ5* and *Δ6* desaturases) apparently allocated to *Fads2-type* genes. To determine the timing of *Fads1-Δ5* and *Fads2-Δ6* evolution in vertebrates we used a combination of comparative and functional genomics with the analysis of key phylogenetic species. Our data show that *Fads1* and *Fads2* genes with *Δ5* and *Δ6* activities respectively, evolved before gnathostome radiation, since the catshark *Scyliorhinus canicula* has functional orthologues of both gene families. Consequently, the loss of *Fads1* in teleosts is a secondary episode, while the existence of *Δ5* activities in the same group most likely occurred through independent mutations into *Fads2* type genes. Unexpectedly, we also establish that events of *Fads1* gene expansion have taken place in birds and reptiles. Finally, a fourth *Fads* gene (*Fads4*) was found with an exclusive occurrence in mammalian genomes. Our findings enlighten the history of a crucially important gene family in vertebrate fatty acid metabolism and physiology and provide an explanation of how observed lineage-specific gene duplications, losses and diversifications might be linked to habitat-specific food web structures in different environments and over geological timescales.

## Introduction

**Figure 1:**
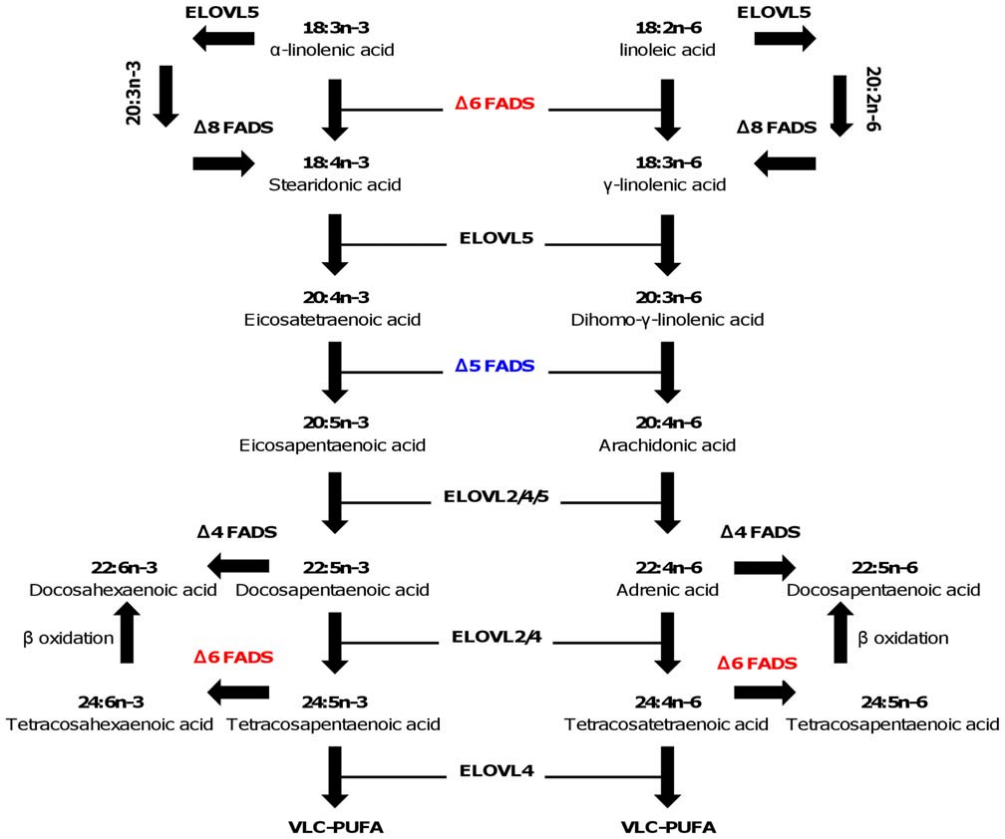
Biosynthetic pathway of very long chain-PUFA (VLC-PUFA).

Fish, like mammals and probably all other vertebrates, are unable to endogenously synthesize polyunsaturated fatty acids (PUFA) and so these compounds are required in the diet [1]. This requirement is met by the basic essential fatty acids, 18:2n-6 (linoleic acid, LOA) and 18:3n-3 (α-linolenic acid, ALA) which cannot be biosynthesized or interconverted in vertebrates, and so all PUFA are ultimately derived from the primary producers, largely plants [2]. Both LOA and ALA have vital functions in themselves, but they also act as precursors for the long chain-PUFA (LC-PUFA), 20:4n-6 (arachidonic acid, ARA), 20:5n-3 (eicosapentaenoic acid, EPA) and 22:6n-3 (docosahexaenoic acid, DHA) [1], [2]. LC-PUFA are essential components of cell membranes, particularly in neural tissues, and they can generally be produced from dietary LOA and ALA in mammals, although evidence suggests that the biosynthesis of EPA and, particularly, DHA from ALA is very low in humans [3], and non-existent in high carnivores such as cats [4]. The biosynthesis of ARA and EPA from LOA and ALA, respectively, involves an initial *Δ6* desaturation, followed by chain elongation, and a further *Δ5* desaturation [5]. An alternative pathway to produce ARA and EPA from LOA and ALA involves an initial elongation and a subsequent *Δ8* desaturation (Figure 1). It has been shown that the same enzyme protein displayed both *Δ6 and Δ8* desaturation activities [6], [7]. It has been is generally accepted that biosynthesis of DHA from EPA in vertebrates requires two further elongation steps and *Δ6* desaturation followed by a peroxisomal chain-shortening step [8] (Figure 1). The *Δ5* and *Δ6/8* enzymes are commonly termed “front-end” desaturases since they introduce the double bound by “counting” from the carboxyl end of the fatty acid molecule. They are encoded by genes denominated fatty acyl desaturases (*Fads*).

Functional *Δ5* and *Δ6* desaturases are found in a wide array of lineages including fungi, invertebrate protostomes and vertebrates [9], [10]. On the basis of functional criteria, *Δ5* genes are thought to have evolved from a *Δ6* ancestor, since the action of the latter provides the substrate for *Δ5* desaturase in the LC-PUFA biosynthesis pathway [9] (Figure 1). However, the evolution of desaturase gene lineages and their respective specificities is far from understood. In humans the *Δ5* and *Δ6* desaturations are catalysed by the products of *FADS1* and *FADS2* genes, respectively. These are organized into a tight physical cluster in chromosome 11 [11]. An additional desaturase, *FADS3*, is also part of the cluster, although its function has not yet been elucidated [11], [12]. In the nematode *Caenorhabditis elegans*, *Δ5* and *Δ6* desaturation activities are also encoded by different *Fads-like* genes, which are found in close proximity in chromosome IV [13], [14]. Nevertheless, the nematode genes are more similar to each other than to any of the mammalian gene set. Furthermore, sequence phylogenies demonstrate that the fungi and nematode *Fads* genes are basal to the human *Fads1*, *Fads2* and *Fads3* clade [9], [10], [13], [14]. This evolutionary pattern of gene orthology *versus* gene function indicates that the acquisition of separate functional *Δ5* and *Δ6* genes occurred at least twice in evolution.

In vertebrate species, a complex evolutionary scenario is also emerging. The pattern reported in mammals is not similar to that found in teleosts. A combination of gene isolation and functional characterization has uncovered at least three distinct organizations in fish. A single bifunctional *Δ5/Δ6* desaturase was described in zebrafish (*Danio rerio*), though this gene is more similar to the mammalian *Fads2* [15]. A similar result was recently described in the rabbitfish (*Siganus canaliculatus*), a marine teleost from which two *Fads*2-related genes have been characterised, one having *Δ5*/*Δ6* activity and the other with *Δ5*/*Δ4* function [16]. Separate *Δ5* and *Δ6* genes were found in Atlantic salmon (*Salmo salar*), and again these are both of the *Fads2-*type [17], [18], [19]. Finally, in most other studied teleost species a single *Δ6* Fads2-like gene and enzyme has been characterized [2], [20], [21], [22].

In summary, *Fads1-Δ5* appears to be mammalian specific, while teleosts have typically *Fads2-Δ6*, with some examples of bifunctional desaturases (e.g. zebrafish) or separate *Fads2-like-Δ5* and *Fads2-like-Δ6* (e.g. Atlantic salmon). Since the LC-PUFA products of “front-end” desaturases, particularly ARA and DHA, are essential components of complex vertebrate nervous systems, the key question is when in vertebrate history did the *Fads1-Δ5* and *Fads2-Δ6* linked activities evolve, and what is the diversity of the *Fads* gene portfolio in vertebrate classes.

At least two evolutionary scenarios can explain the described pattern of gene diversity versus *FADS* selectivity in vertebrate classes. First, *Fads1-Δ5* and *Fads2-Δ6* may have duplicated just before mammalian speciation. In that case, the phylogenetic analysis, which strongly groups teleost *Fads* genes with mammalian *Fads2*, is difficult to interpret unambiguously. An alternative explanation indicates that the duplication event generating both gene lineages, *Fads1* and *Fads2*, is older as is the respective *Δ5* and *Δ6*-linked activities. In this case, teleosts would have lost the *Fads1-Δ5* lineage, while in some species the *Δ5* function was regained through duplication/diversification of a *Fads2-Δ6* ancestor. To test these hypotheses we took two approaches. First, we determined the diversity and evolutionary history of *Fads* genes in various vertebrate classes where full genomes are available (teleosts, amphibians, birds, reptiles and mammals). Secondly, we isolated and characterized the *Fads* gene family in the basal gnathostome, the cartilaginous fish small-spotted catshark (*Scyliorhinus canicula*), formerly commonly known as the lesser spotted dogfish. Our findings show that clear orthologues of *Fads1* and *Fads2* arrived before gnathostome radiation. Furthermore, functional analysis demonstrates that the dogfish *Fads1* and *Fads2* are *Δ5* and *Δ6* desaturases, respectively. Thus, a complete *Fads1* loss is observed in teleosts, with the occasional recruitment of the *Δ5* function into *Fads2* genes. Interestingly, we also find that some teleost species have completely lost the full set of “front-end” desaturases (e.g. the pufferfishes *Takifugu rubripes* and *Tetraodon nigroviridis*). Finally, a novel Fads gene was uncovered in some mammalian species, while in birds and reptiles the *Fads1-like* portfolio has specifically expanded, with yet unknown functional consequences. We argue that the overall *Fads* gene/function history in vertebrates involves selective dietary/nutritional pressures, combined with gene events of gain and loss.

## Results

### The Fads gene portfolio has different complements in vertebrate classes

To investigate the portfolio of *Fads* genes in vertebrate classes we searched the genomes of human, Rhesus macaque, mouse, dog, rabbit, opossum, platypus, green anole, chicken, western clawed frog, zebrafish, medaka, stickleback, pufferfish and green-spotted puffer (Table S1). This task involved the identification of genome annotated sequences but also direct searches through the Blastp algorithm (PSI Blast). In humans we found the previously described *FADS1*, *FADS2* and *FADS3* genes in chromosome 11 (Table S1). A similar *Fads* gene set was also uncovered in the opossum and the dog, though the *Fads2* sequence in opossum was shorter due to the presence of a sequence genome gap that disallowed its analysis. In the Rhesus macaque, rabbit, mouse and platypus, apart from the classic desaturase gene set, a fourth *Fads*-like gene was found. This sequence contains the common *FADS* features such as the presence of three conserved histidine motifs (Figure S1). Birds and reptiles showed the highest number of *Fads* gene ORFs of the analysed species (Table S1), with the presence of four genes in the chicken and six in the green anole. The amphibian *X. tropicalis* has two clear *Fads* genes, while a third *Fads-like* sequence was found in a distinct scaffold, but was incomplete and could not be included in the analysis. In teleosts, several different scenarios were noted. The zebrafish has a single *Fads* gene as previously described [15], while none were found in the two pufferfish species (Table S1). In stickleback, three *Fads-like* gene annotations are present at distinct genomic regions. Nevertheless, examination of protein and nucleotide sequence (introns included) showed that they correspond to the exact same sequence and are most likely the result of poor genome assembly in the region. Three medaka *Fads-like* annotations were also uncovered, but two sequences were incomplete with several missing exons. Using the human *FADS2* genomic organization and the genome sequence of the medaka we were able to deduce the complete sequence of the two extra genes (Figure S2). We conclude that there are three *Fads-like* genes in medaka. Finally, a single copy *Fads* gene was found in the invertebrate chordate amphioxus.

**Figure 2:**
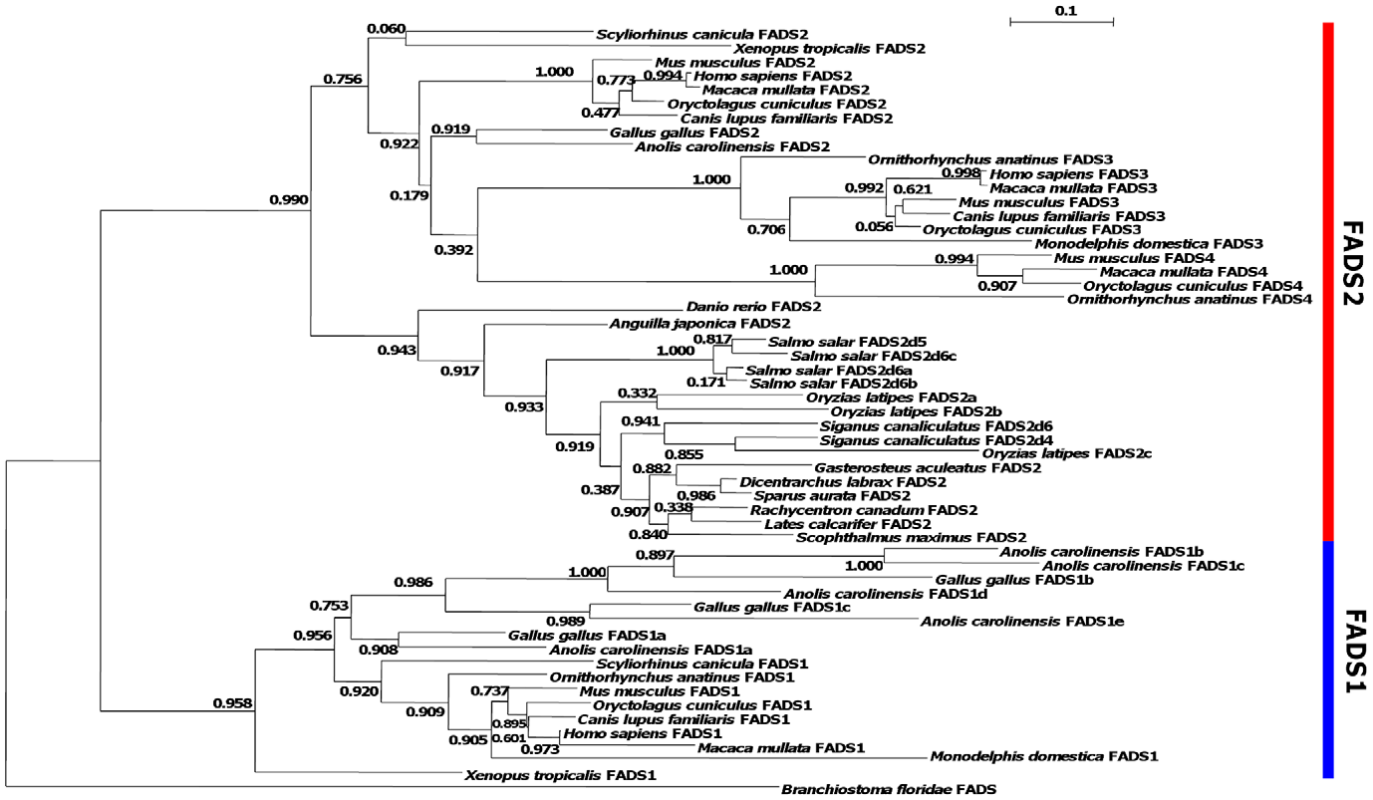
Maximum likelihood tree for the *Fads* gene family based on protein sequences. aLRT values are shown on each node. Accession numbers of the sequences are given in table S1.

The search of *Fads front-end* desaturases in vertebrate genomes uncovered an unexpected diversity of genes. To model the relative timing of duplication and evolutionary histories, we next examined their phylogenetic relationships (Figure 2). Apart from the desaturases identified above, we also included other reported *Fads* sequences in particular from teleosts. Two well-supported clades can be observed, one including *Fads1-like* sequences (aLRT 0.958) and a second with *Fads2*, *Fads3* and the novel mammal-specific gene family which we name *Fads4* (aLRT 0.990). The *Fads1* clade includes sequences from tetrapod species but not teleosts. Furthermore, the unexpected high number of *Fads* sequences found in reptiles and birds can now be unequivocally attributed to a lineage-specific expansion of the *Fads1* gene group. We labelled each bird and reptile species gene (*a* to *e*) according to their position in the cluster (see below). In the *Fads2/3/4* clade we find that mammalian species have single copy genes of *Fads2*, *Fads3* and *Fads4*, though the entire group does not form an independent well supported clade. *Fads3* and *Fads4* are outgrouped by the chicken and anolis *Fads2* but this is a poorly supported clade. This larger collection is outgrouped by the amphibian and teleost FADS2 genes. We opted to maintain the gene nomenclature, although the mammalian *Fads2*, *Fads3* and *Fads4* are probably co-orthologues of the remaining *Fads2* sequences. Though some teleost species have more than one *Fads* gene these have clearly resulted from species-specific expansions as well (e.g. Atlantic salmon). Thus, the present phylogenetic tree suggests that teleosts have probably lost a *Fads1* orthologue; an expansion of a *Fads1-like* gene took place in the ancestor of birds and reptiles; and the *Fads2/3/4* duplications are specific to mammals.

**Figure 3:**
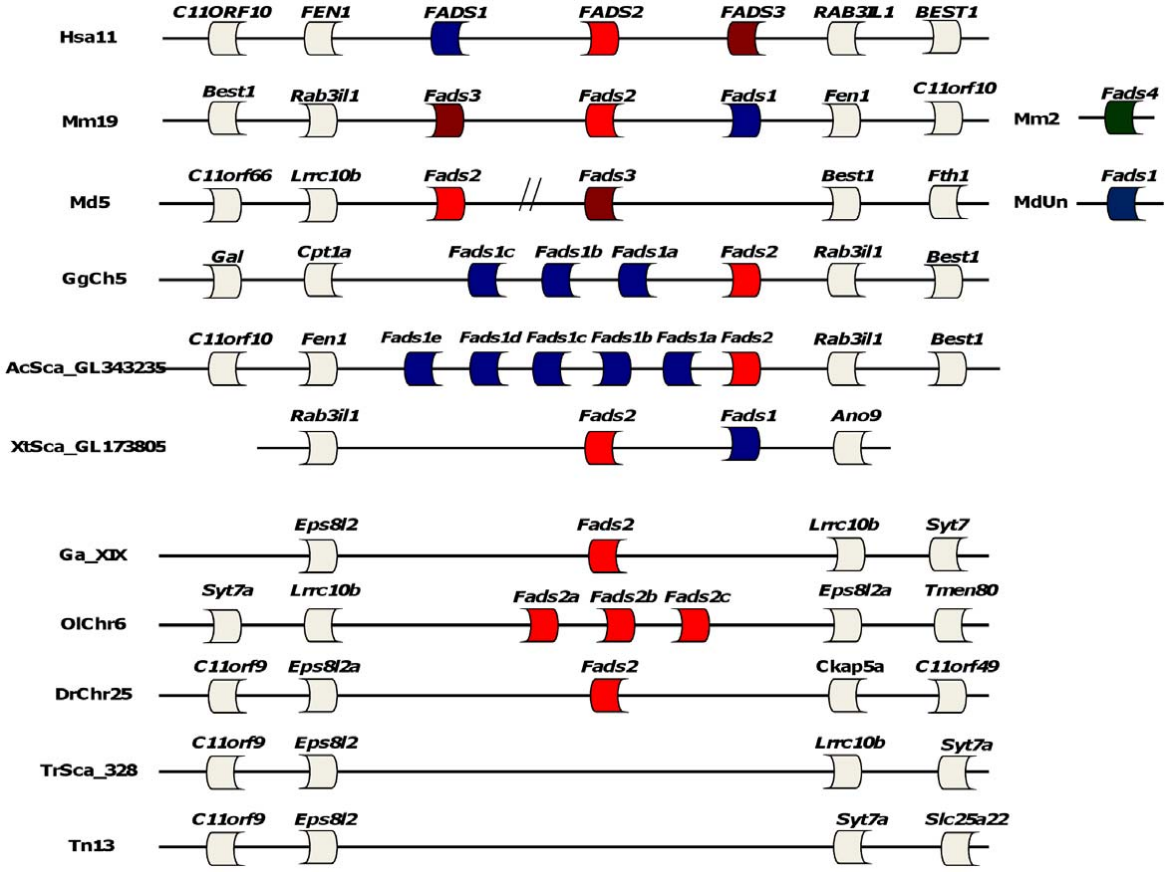
Synteny map of the *Fads* gene cluster in vertebrates. Mammals (Hs-*Homo sapiens*, Mm-*Mus musculus*, Md-*Monodelphis domestica*), birds (Gg-*Gallus gallus*), reptiles (Ac-*Anolis carolinensis*), amphibians (Xt-*Xenopus tropicalis*) and teleosts (Ga-*Gasterosteus aculeatus*, Ol-*Oryzias latipes*, Dr-*Danio rerio*, Tr-*Takifugu rubripes*, Tn-*Tetraodon nigroviridis*). Double dash denotes gap.

The human “front-end” desaturase gene set is organized into a tight physical cluster in chromosome 11 [11]. To determine whether this represents a distinctive feature in the desaturase genomic organization, we analyzed the location of *Fads* genes in the various species (Figure 3). Synteny analysis can also assist in the discrimination between gene loss or incomplete genome sequence as causes for gene absence, such as we have shown in the pufferfishes, *T. rubripes* and *T. nigroviridis*.

In tetrapods, the *Fads* gene(s) *locus* arrangement is highly conserved with most of the flanking gene families being shared. One exception is found with the opossum *Fads1* that maps at a distinct location (Figure 3). However, the presence of a gap between *Fads2* and *Fads3* precludes a final conclusion regarding the conservation of this gene cluster. The mouse *Fads4* also maps at a different chromosome, a condition which is repeated in all the mammalian species having *Fads4* (not shown). Therefore, this duplication probably involved a parallel translocation into a distinct genomic region. In teleosts, although the flanking gene families are different from those observed in the tetrapod *Fads* locus, their human orthologues map to chromosome 11. For example, *Eps8l1* which flanks teleost *Fads* genes is also found at the human chromosome 11 (though distantly), while *Lrcc10b* and *Syt7* orthologues map very close to the human *Fads* gene cluster. The genomic *loci* investigation served also to address the failure to encounter *Fads* genes in the pufferfishes. While *Fads* genes are not found in these species, we find that this is not the result of an incomplete genome sequence, but gene deletion. Thus, we find that genes typically bordering the teleosts *Fads* gene(s) have a similar arrangement in pufferfishes, but without any intervening *Fads* ORF (Figure 3).

### Cartilaginous fish have clear functional orthologues of Fads1 and Fads2/3/4

Our phylogenetic analysis suggests that the duplication timing of *Fads1* and *Fads2/3/4* pre-dates the divergence of teleosts and tetrapods, although teleosts have no *Fads1* orthologue. Consequently, *Fads1* and *Fads2/3/4* gene lineages are presumably older than anticipated. To better define the timing of the *Fads1*/*Fads2* duplication event, we examined the gene complement in an earlier diverging branch of the vertebrates, the chondrichthyans. Through PCR with degenerate primers and RACE PCR we obtained two distinct full coding sequences of “front-end” desaturase genes. Both have all the distinctive features of *Fads* genes, namely the histidine boxes (Figure S1). To address the orthology of the new sequences found in catshark (*S. canicula*), we undertook phylogenetic analysis (Figure 2). We found that one of the catshark genes robustly groups within the *Fads1* tetrapod group (though not basally as would be expected), while the other gene is basal to the mammalian and sauropsid *Fads2* clade, together with the amphibian *Fads2* orthologue. Thus, we named these *ScaFads1* and *ScaFads2*, respectively. Whether this represents the full complement of *Fads* gene in the catshark is at present unknown. However, BLAST to the genome of a second chondrichthyan species, the elephant shark (*C. milii*), also found only partial segments with high similarity to *ScaFads1* and *ScaFads2* (not shown). Together these results show that *Fads1* and *Fads2/3/4* ancestors were present prior to the emergence of gnathostomes, and they indicate the loss of *Fads1* in the teleost clade.

**Figure 4:**
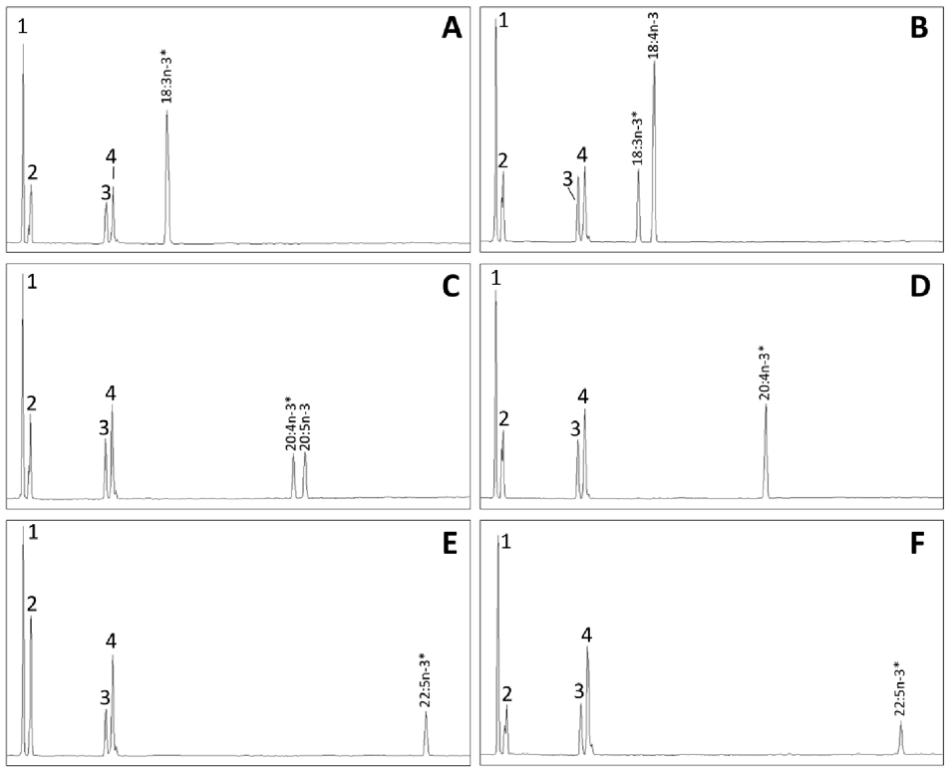
Functional characterization of the newly cloned *Scyliorhinus canicula* fatty acyl desaturases FADS1. (Panels A, C and E) and FADS2 (panels B, D and F) in transgenic yeast (*Saccharomyces cerevisiae*) grown in the presence of Δ6 substrates 18:3n-3 (A and B), Δ5 substrates 20:4n-3 (C and D) and Δ4 substrates (E and F). Fatty acids were extracted from yeast transformed with pYES2 vector containing the ORF of the putative fatty acyl desaturase cDNA as an insert. The first four peaks in all panels are the main endogenous fatty acids of *S. cerevisiae*, namely 16:0 (1), 16:1 isomers (2), 18:0 (3), and 18:1n-9 (4). Substrates (“*”) and their corresponding desaturated products are indicated accordingly in panels A–F. Vertical axis, FID response; horizontal axis, retention time.

In mammals, FADS1 is a *Δ5* enzyme, while FADS2 is a *Δ6* desaturase. In contrast, in teleosts the exclusive *FADS2* portfolio has diverse desaturase activities, including *Δ5*. Thus, we next performed functional characterization of the FADS proteins from catshark, by determining the fatty acid profiles of yeast transformed with *ScaFads1* and *ScaFads2* constructs (Figure 4). Transgenic yeast were grown in the presence of potential fatty acid substrates for *Δ6*, *Δ5* and *Δ4*. The GC-MS analysis clearly indicated that yeast transformed with *ScaFads1* was able to bioconvert the *Δ5*-desaturase substrates 20:4n-3 and 20:3n-6 to 20:5n-3 and 20:4n-6, respectively, with no activity observed as *Δ6-* and *Δ4-*desaturase (Figure 4; Table 1). Yeast expressing *ScaFads2* actively transformed the *Δ6*-desaturase substrates 18:3n-3 and 18:2n-6 producing 18:4n-3 and 18:3n-6, respectively, with no activity detected towards *Δ5-* and *Δ4-*desaturase substrates (Figure 4; Table 1). Both *ScaFads1* and *ScaFAds2* gene products exhibited higher activity towards n-3 PUFA substrates than the corresponding n-6 PUFA substrates (Table 1).

**Table 1:** Functional characterization of catshark *Scyliorhinus canicula FADS1* and *FADS2* proteins in *Saccharomyces cerevisiae*.

FA substrate	Product	Conversion (%)		Activity
		FADS1	FADS2	
18:3n-3	18:4n-3	0	73	Δ6
18:2n-6	18:3n-6	0	57	Δ6
20:4n-3	20:5n-3	55	0	Δ5
20:3n-6	20:4n-6	29	0	Δ5
22:5n-3	22:6n-3	0	0	Δ4
22:4n-6	22:5n-6	0	0	Δ4

Results are expressed as a percentage of total fatty acid substrate converted to desaturated product.

## Discussion

**Figure 5:**
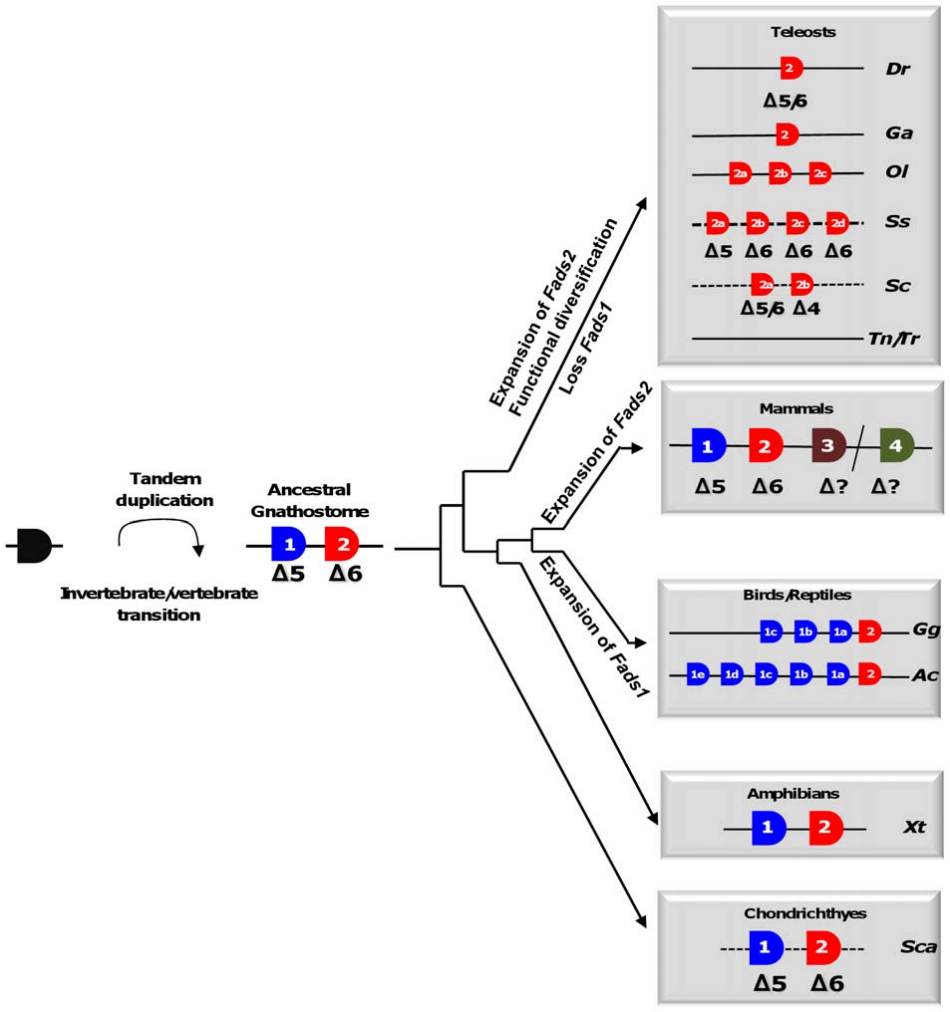
Evolutionary model of *Fads* gene diversification along the vertebrate lineage. Dotted line indicates physical linkage unknown. Gg-*Gallus gallus*, Ac-*Anolis carolinensis*, Xt-*Xenopus tropicalis*, Ga-*Gasterosteus aculeatus*, Ol-*Oryzias latipes*, Dr-*Danio rerio*, Tr-*Takifugu rubripes*, Tn-*Tetraodon nigroviridis*, Ss- *Salmo salar*, Sc- *Siganus canaliculatus*, and Sca- *Scyliorhinus canicula*.

In this work we aimed to elucidate some aspects of the evolutionary history and function of *Fads* genes in vertebrates. This gene family participates in the fundamental biochemical pathway of LC-PUFA biosynthesis (Figure 1). By taking a comparative genomics approach with the investigation of key phylogenetic species, we attempted to resolve the question of when in vertebrate evolution did *Fads1-Δ5* and *Fads2-Δ6* genes evolve, and whether the gene diversity is wider than previously anticipated. Our results support an evolutionary scenario that combines processes of gene duplication, gene loss and functional diversification (Figure 5).

We identified three noteworthy events of gene duplication. The isolation of clear *Fads1* and *Fads2/3/4* orthologues in the catshark indicates that the first duplication episode from an ancestral *Fads* occurred prior to the gnathostome radiation, but after invertebrate chordate divergence (Figure 5). However, based on the current evidence we cannot conclude whether this happened before or after the lamprey-gnathostome split. Although, a search of the sea lamprey (*Petromyzon marinus*) genome found a single incomplete desaturase sequence, this was unsuitable for robust phylogenetic analysis (not shown). Although our data is equivocal regarding the duplication timing of *Fads2/3/4* (since the mammalian genes do not form an independent statistically supported group), we find more parsimonious that a single *Fads2* gene duplicated in the ancestor of mammals to originate *Fads2*, *Fads3*, and *Fads4*. Otherwise numerous events of gene loss would have to be taken into account.. Therefore, these genes are probably co-orthologues of the *Fads2* from other vertebrate classes. However, a stable *Fads* gene set was not retained in mammals as a whole. *Fads4* was lost in various species including humans, dog and the opossum. Also, we find that the well-known very limited capacity for LC-PUFA biosynthesis observed in some extreme carnivores such as cats [4] is probably related with gene loss. Although the genome sequence is incomplete, we found partial *Fads1* and *Fads4* sequences in the cat genome, but no orthologues of *Fads2* or *Fads3* (L Filipe C Castro, unpublished). These findings are consistent with previous biochemical characterizations, in particular, the apparent absence of *Δ6* activity in comparison to presence of *Δ5* activity in domestic cat (*Felix catus*) [23], [24]. In the tetrapod clade, we demonstrated that a large and unique independent expansion of the *Fads1* lineage in chicken (three genes) and green anole (five genes) has taken place but, at present, with no clues on possible functional impacts (Figure 5).

The examination of catshark desaturases shows that the *Fads1-Δ5* and *Fads2-Δ6* activities evolved before gnathostome radiation, and were retained in cartilaginous fish and mammals (and probably in all the tetrapod clade) (Figure 5). The findings in the catshark imply also that *Fads1-Δ5* was lost in the teleost ancestor. Consistently, the vast majority of teleost species examined to date have only *Fads2-Δ6* gene(s) [2], [21], [25]. Nevertheless, although the archetypal *Δ5* desaturase, FADS1, has been lost in teleost ancestry, the *Δ5* activity was selectivity regained within specific teleost lineages. The zebrafish *Fads2* gene, codes for a bifunctional *Δ5*/*Δ6* enzyme [15], and the two *Fads2*-like genes in rabbitfish encode bifunctional *Δ5*/*Δ6* and *Δ4*/*Δ5* enzymes [16]. In Atlantic salmon, *Δ5* activity is encoded by a *Fads2-type* gene that shows >95% deduced amino acid identity with the salmon *Δ6* having arisen from a salmonid-specific duplication [17], [18], [19]. Thus, the various teleost species with *Fads2-Δ5* represent secondary and lineage specific events. These teleost lineage-specific events and the resulting *Fads* functional plasticities parallel a similar scenario to that observed in the invertebrate protostome *C. elegans* [14]. Here, two separate *Fads-Δ5/Fads-Δ6* genes are also present and have probably evolved from a *Δ6* ancestor [9]. This interchange of functions between *Δ6* to *Δ5* represents an unusual case of functional plasticity. Theoretically, the capacity to change desaturase specificity in this way must be dependent on a minimal number of mutations. Otherwise, re-evolution of a *Δ5* phenotype on a typically *Δ6* genotype (*Fads2*) would be probabilistically unlikely. At present, no study has yet detailed the crucial domains or amino acid residues of animal *FADS* proteins responsible for their catalytic properties. However, in the ancestrally related *Δ9* desaturase enzyme, coded by Stearoyl CoA Desaturase (*Scd*), a recent study has discriminated three single amino acids as responsible for change of specificities [26]. We would predict a similar behaviour in the case of *FADS* desaturases.

It has been suggested that the teleost *Fads* gene(s) portfolio and their functional activities have been modelled by habitat-specific LC-PUFA abundance [1], [2], [27]. Thus, species, for example zebrafish and Atlantic salmon, inhabiting some freshwater environments have diversified their gene and catalytic complement in response to the lack or reduced levels of LC-PUFA in their natural diets [25]. This lack of LC-PUFA is also evident in terrestrial habitats that are fuelled by green plants [27], [28], and is an explanation for the retention of both *Δ5* and *Δ6* desaturase genes in terrestrial tetrapods. In contrast, carnivorous marine and eutrophic freshwater species would have no need for endogenous synthesis of LC-PUFA, given the luxus of diatom and dinoflagellate-derived LC-PUFA [25], [27], [28]. In this regard, two groups of marine fish are particularly interesting. In the case of the pufferfish *T. rubripes* and *T. nigroviridis*, a complete absence of “front-end” desaturase genes is observed, which we would argue results from very low environmental pressure to maintain *Δ6* and *Δ5* desaturase genes. In contrast the marine species rabbitfish has duplicated and diversified *Fads2*-like genes, resulting in a unique diversity of desaturase specificity [16]. This might be explained by the fact that this species has specialised in feeding on seagrasses, a group of marine flowering plants including eelgrass, which have evolved from terrestrial plants that recolonized the sea and, like terrestrial plants, lack LC-PUFA [29].

Despite the exceptions discussed above it is clear that the vast number of teleosts examined to date retain a *Fads2*-*Δ6* even if they inhabit marine environments. Although *Δ6* and *Δ5* activities are interdependent in the pathway of EPA biosynthesis, *Δ6* activity is also required for the production of DHA from EPA whereas *Δ5* is not and so retention of *Δ6* may be due to specific biochemical/metabolic/physiological factors albeit still related to dietary supply of LC-PUFA. Comparative analysis of *Δ6* gene expression between salmonids and marine fish (e.g. Atlantic cod, cobia, Asian sea bass) shows some remarkable differences. While the highest levels of *Fads2-Δ6* expression and desaturation activity are in liver and intestine in salmon, the *Fads2-Δ6* expression and desaturation activity is low in marine fish liver and intestine, but high in the brain [30] (Tocher et al., 2006), which, as with all neural tissues, has a fatty acid composition with a very high DHA:EPA ratio [1]. Thus, it has been suggested that retention *Fads2-Δ6* in marine fish may be to maintain membrane DHA levels (by metabolism of EPA) in neural tissues at times of high physiological demand including during embryonic and larval development [20], [22], [30]. Despite the abundance of LC-PUFA in the marine environment and likely in the natural diet of the catshark, we could speculate that a functionally similar role, that is the ability to precisely regulate tissue DHA:EPA ratios, may explain the retention of *Δ6* enzymes. However, the high levels of EPA and DHA in the present marine environment would make the retention of *Δ5* desaturase activity more difficult to account for, since, according to the arguments above, there would be little pressure and consequently no requirement for endogenous LC-PUFA biosynthesis in marine habitats. However it is well known that that the early ocean, in which the basal gnathostomes evolved, was a very different habitat to that we see today. Particularly relevant is the paleontological evidence indicating that the major LC-PUFA producing phytoplankton, the diatoms and dinoflagellates, did not come to prominence until the Triassic (i.e. not more than 250 Ma ago) [31]. Prior to this primary productivity in the early oceans appears to have dominated by green algae and cyanobacteria. Since the appearance of sharks occurred more than 400 Ma years ago, predating the emergence of diatoms and dinoflagellates, the acquisition of both *Fads1* and *Fads2* genes may have been necessary to enable survival in a relatively LC-PUFA-poor ecosystem. In contrast the appearance and subsequent enormous radiation of the Teleostei is coincident with the rise to domination of the diatoms and dinoflagellates.

The appearance of morphological and physiological innovations in the vertebrate clade has long been linked with gene duplication, namely genome duplications [32]. Two rounds of genome duplications (2R) are now an established event in vertebrate evolution [33]. The vertebrate *Fads* evolutionary pattern indicates that an increase in gene number took place just before the emergence of vertebrates. In effect, we find that a tandem gene duplication of *Fads* gene precursor originated separate *Δ5* and *Δ6* genes, with further genes increases in particular lineages. Our analysis of the *Fads* history shows that the increase in number of the *front-end* desaturase vertebrate set was linked with tandem duplications and not 2R. This observation contrasts with what is found in other desaturase gene families. The *Scd* genes, which catalyze the desaturation of saturated fatty acyl-CoA substrates at the *Δ9* position, were recently showed to have specifically duplicated in the vertebrate lineage as a result of 2R [34], [35].

A discussion of the LC-PUFA biosynthetic pathway cannot be complete without considering the other main enzymatic steps, those performed by fatty acid elongases, *Elovl2* and *Elovl5* (Figure 1). The partial or complete loss of front-end desaturases in teleost species indicates the disruption of the LC-PUFA synthesis pathway (Figure 1). For example, absence of a *Δ5* gene implies the inability to synthesize either EPA or ARA from 20:4n-3 and 20:3n-6 substrates, respectively. Curiously, the presence of *Fads2-Δ5* genes in teleosts is apparently correlated with the retention *Elovl2* orthologues (e.g. zebrafish and Atlantic salmon) [36], [37]. In contrast, medaka, stickleback, fugu and pufferfish have no obvious *Elovl2* sequences [36]. Although the *Fads2* genes from these species have not yet been functionally characterized it is tempting to suggest that they are functionally *Δ6*. This apparent correlation between the genome complement of desaturases and elongases genes would imply that cartilaginous fish should have also an *Elovl2* since we have now shown the presence of a *Fads1- Δ5* gene. Searches to the elephant shark genome confirm that *Elovl2* like sequences are present (not shown). Exactly why teleosts that possess no desaturase gene complement still retained an *Elovl5* and *Elovl4* such as *T. rubripes* and *T. nigroviridis* remains to be investigated.

In summary, we provide a clear framework of *“front-end”* desaturase evolution in vertebrate history. We present an explanation of how observed lineage-specific gene duplications, losses and diversifications might be linked to habitat-specific food web structures in different environments and over geological timescales.

## Materials and Methods

The animals used in the research described in this paper were treated in accordance with the Portuguese Animals and Welfare Law (Decreto-Lei n° 197/96) approved by the Portuguese Parliament in 1996. Institutional animal approval by CIIMAR/UP and DGV (Ministry of Agriculture) was granted for this study.

### Mining of FADS genes, phylogenetic and synteny analysis

The *Fads* gene portfolio was identified in release 63 of the Ensembl database (www.ensembl.org) and Genbank from the following species: *Homo sapiens* (human-placental mammal), *Macaca mullata* (Rhesus macaque-placental mammal), *Mus musculus* (mouse-placental mammal), *Canis familiaris* (dog-placental mammal), *Oryctolagus cuniculus* (rabbit-placental mammal), *Monodelphis domestica* (opossum-marsupial), *Ornithorhynchus anatinus* (platypus-monotreme), *Anolis carolinensis* (green anole-reptile), *Gallus gallus* (chicken-bird), *Xenopus tropicalis* (western clawed frog-amphibian), *D. rerio* (teleost), *Gasterosteus aculeatus* (three-spined stickleback-teleost), *Oryzias latipes* (medaka-teleost), *T. nigroviridis* (green-spotted pufferfish-teleost), *T. rubripes* (‘Fugu’ pufferfish-teleost), and *Branchiostoma floridae* (amphioxus-cephalochordate). The search of the cartilaginous fish *Callorhinchus milii* (elephant shark-chondrichthyes) genome was performed using the human *FADS1* and *FADS2* sequences with TBLASTN at http://esharkgenome.imcb.a-star.edu.sg/Blast/ [38].

To identify sequences that were poorly annotated or non-annotated in databases we also performed a BLAST search Blastp (PSI-BLAST). Some sequences were manually curated using the human gene *FADS1* or *FADS2* gene structure as a model.

Protein sequences from *Fads* genes were aligned using MAFFT (http://mafft.cbrc.jp/alignment/server/) with the L-INS-i method [39]. The alignment was further adjusted manually with gap removal. The final dataset had a total of 55 sequences and 297 characters. For tree reconstruction, we first applied ProtTest [40] to estimate the optimal model of amino acid substitution (JTT+I+G+F). A maximum likelihood tree was constructed using PHYML (online) [41] with the JTT model. The amino acid frequency (equilibrium frequency), proportion of invariable sites and gamma-shape (4 rate substitution categories) for the amino acid substitution rate heterogeneity parameters were estimated from the dataset. Confidence in each node was assessed by aLRT non-parametric branch support (SH-like) [42]. The phylogenetic tree was rooted with the amphioxus sequence using NJPlot (version 2.3) (pbil.univ-lyon1.fr/software/njplot.html) [43].

Genomic regions containing *Fads* genes were identified in Ensembl and Genbank databases. The two closest gene families flanking *Fads* genes were identified.

### Cloning and sequencing of the S. canicula Fads1 and Fads2 cDNA

Catshark specimens in May 2010 were given an overdose of tricane methylsulfonate (1∶5000 MS-222 Aquapharm UK) and killed by cervical transection. Total RNA was isolated from the collected tissues using the Illustra RNAspin minikit from GE Healthcare (Little Chalfont, UK) with on-column DNase I treatment. RNA concentration was measured with a Qubit fluorometer platform (Invitrogen, Carlsbad CA). Conversion of total RNA into first strand cDNA was performed using the iScript cDNA synthesis following the manufacturer recommendations (Bio-Rad). Polymerase chain reaction (PCR) used cDNA from catshark (*S. canicula*) and degenerate primers for *Fads1* and *Fads2* (Table S2). Rapid amplification of cDNA ends (RACE) PCR was used to obtain the full open reading frames (ORF) from the original sequence fragments (Clontech, USA). For all PCR protocols, the Phusion Flash hot start high fidelity polymerase mix with the manufacture recommended conditions was used (Finnzyme, Helsinki FI). Amplicons of the appropriate size were isolated from the agarose gel (GFX cleaning kit, GE Healthcare), and sequenced directly with one of the flanking primers (Stabvida, Portugal).

### Functional assay of the Fads1 and Fads2 in S. canicula

Primers with restriction sites of *Hin*dIII and *Eco*RI flanking the predicted longest ORF of *ScaFads1* and *ScaFads2* were used for PCR with Phusion Flash hot start high fidelity polymerase mix (Table S2). Bands of the appropriate size were cloned into pGEMT-easy. Four clones were sequenced and compared with the cDNA assembled contig. After restriction digest, the insert was cloned into pYES2 yeast expression vector (Invitrogen), and sequenced. The constructs pYES2-*ScaFads1* and pYES2-*ScaFads2* were transformed into *Saccharomyces cerevisiae* competent cells (strain InvSc, Invitrogen). Transformation and selection of yeast with recombinant pYES2 plasmids and yeast culture and fatty acid (FA) analyses were performed as described in detail previously [15], [44]. Transgenic yeast were grown in the presence of potential fatty acid substrates for *Δ6* (18:3n-3 and 18:2n-6), *Δ5* (20:4n-3 and 20:3n-6) or *Δ4* (22:5n-3 and 22:4n-6) activities. The FA were added to the yeast cultures at final concentrations of 0.5 (C18), 0.75 (C20) and 1.0 (C22) mM as uptake efficiency decreases with increasing chain length. Yeast transformed with empty pYES2 were also grown in presence of PUFA substrates as control treatments. After 2-days culture at 30°C, yeast were harvested and washed, and lipid extracted by homogenization in chloroform/methanol (2∶1, v/v) containing 0.01% butylated hydroxy toluene as antioxidant. Methyl esters of FA were prepared, extracted, purified, and analyzed by GC in order to calculate the proportion of substrate FA converted to desaturated FA product as [product area/(product area+substrate area)]×100. Identities of FA peaks were based on GC retention times and confirmed by GC-MS as described previously [15], [16].

## Supporting Information

Figure S1 **Alignment of amino acid sequences of FADS proteins from** ***H. sapiens*** **(FADS1, FADS2 and FADS3),** ***M. musculus*** **(FADS1, FADS2, FADS3 and FADS4) and** ***S. canicula*** **(FADS1 and FADS2).** The “HPGG” characteristic of cytochrome *b5* domain is underlined in black. The three conserved histidine motifs “HXXXH”, “HXXHH”, and “QXXHH” is underlined in red.
(DOCX)

Figure S2 ***Fads2b*** **and** ***Fads2c*** **intron-exon organization in** ***Oryzias latipes*** **.**
(DOCX)

Table S1 **List of identified Fads sequences and the accession numbers for all the sequences used in the phylogenetic analysis.**
(DOC)

Table S2 **List of primers used to isolate and characterize** ***Fads1*** **and** ***Fads2*** **genes in** ***Scyliorhinus canicula*** **.**
(DOC)
