# Supplementary material for: Functional Desaturase Fads1 (Δ5) and Fads2 (Δ6) Orthologues Evolved before the Origin of Jawed Vertebrates
Source: PLoS One. 2012 Feb 22;7(2):e31950. doi: 10.1371/journal.pone.0031950 (PMC3285190; doi:10.1371/journal.pone.0031950)
Supplement: Figure S1 — Alignment of amino acid sequences of FADS proteins from H. sapiens (FADS1, FADS2 and FADS3), M. musculus (FADS1, FADS2, FADS3 and FADS4) and S. canicula (FADS1 and FADS2). The “HPGG” characteristic of cytochrome b5 domain is underlined in black. The three conserved histidine motifs “HXXXH”, “HXXHH”, and “QXXHH” is underlined in red. (DOCX) [file pone.0031950.s001.docx]

10 20 30 40 50 60 70 80 90 100

....|....|....|....|....|....|....|....|....|....|....|....|....|....|....|....|....|....|....|....|

**HsFADS1**  **MGTRAARPAGLPCGAENPARRRLALGARQQIHSWSPRTPSTRLTAPAGPARGVARPAMAPDPVAA-------ETAAQGPTPRYFTWDEVAQRSGCE-ERW**

**HsFADS2**  **---------------------------------------------------------.GKGGNQG------EGA.EREVSVPT.S.E.IQKHNLRT-D..**

**HsFADS3**  **---------------------------------------------------------.GGVGEPG----PREGP.QP.APLPT.C.EQIRAHDQPG-DK.**

**MmFADS1**  **---------------------------------------------------------......PTP-----GPAS..LRQT.....E.......R.K...**

**MmFADS2**  **---------------------------------------------------------.GKGGNQG------EGSTERQAPMPT.R.E.IQKHNLRT-D..**

**MmFADS3**  **---------------------------------------------------------.GGVGEPGGGPGPREGP.PL.APLPI.R.EQIR.HDLPG-DK.**

**MmFADS4**  **------------------MKLEEK.EHNESLVGK.RPCLHDTHQ.NGK.IANGNPT.NGKVE.YEK-QEANGKGNRL.KCLNLY..Q.IQRH.QEA-DQ.**

**ScaFADS1**  **---------------------------------------------------------.G--------------SS.ECDRD.F.N.E..QRHCTRS-D..**

**ScaFADS2**  **---------------------------------------------------------.GKGGEKG------E.EGEYEKMLALY..E.IQKHN.KT-DK.**

110 120 130 140 150 160 170 180 190 200

....|....|....|....|....|....|....|....|....|....|....|....|....|....|....|....|....|....|....|....|

**HsFADS1**  **LVIDRKVYNISEFTRRHPGGSRVISHYAGQDATDPFVAFHINKGLVKKYMNSLLIGELSPEQPSFEPTKNKELTDEFRELRATVERMGLMKANHVFFLLY**

**HsFADS2**  **..........TKWSIQ....Q...G....E....A.R...PDLEF.G.FLKP......A..E..QDHG..SKI.ED..A..K.A.D.N.F.T.......L**

**HsFADS3**  **...E.R..D..RWAQ.......L.G.HGAE....A.R...QDLNF.R.FLQP......A..E..QDGPL.AQ.VED..A.HQAA.D.K.FD.SPT..AFL**

**MmFADS1**  **...........D.S................................R...........A............A......................L...V.**

**MmFADS2**  **.........VTKWSQ.....H...G..S.E....A.R...LDLDF.G.FLKP......A..E..LDRG.SSQI.ED..A.KK.A.D.N.F.T..L..F.L**

**MmFADS3**  **...E.R..D..RWAQ.......L.G.HGAE....A.H...QDLHF.R.FLKP......A..E..QDGAQ.AQ.IED..A..QAA.D.K.FE.DTT..A.L**

**MmFADS4**  **.........VTDWAGK....R..LN.........A.R.M.LDL.M..L.LKP.........E..Q.KN..AQ.VED.....K.L.A.NMFS..LR..F.H**

**ScaFADS1**  **...C....D..D.SK.....A...G...........T....D.T..N..LK.......A.D...L.SW.S.PA..D......L...TN.L.P.KL..AGL**

**ScaFADS2**  **.........VTNWALK....I.....S..E....A.Q...PDLIF.R.FLKP......A.GE.NQDRQ.KT..VED..A..K.A.D.K.F.TDM...S..**

210 220 230 240 250 260 270 280 290 300

....|....|....|....|....|....|....|....|....|....|....|....|....|....|....|....|....|....|....|....|

**HsFADS1**  **LLHILLLDGAAWLTLWVFGTSFLPFLLCAVLLSAVQAQAGWLQHDFGHLSVFSTSKWNHLLHHFVIGHLKGAPASWWNHMHFQHHAKPNCFRKDPDINMH**

**HsFADS2**  **.A..IA.ESI..F.VFY..NGWI.T.IT.FV.ATS..........Y.....YRKP.....V.K.........S.N....R.........I.H....V..L**

**HsFADS3**  **.G...AMEVL...LIYLL.PGWV.SA.A.FI.AIS...SWC....L..A.I.KK.W...VAQK..M.Q...FS.H...FR.........I.H....VTVA**

**MmFADS1**  **........V.......I....LV..I.......T..................G..T............................................**

**MmFADS2**  **.S..IVMESL..FI.SY...GWI.T.VT.FV.ATS..........Y.....YKK.I...VV.K.........S.N....R.........I.H.....KSL**

**MmFADS3**  **.G...AMELL...IIYLL.PGWVSSI.A.LI.AIS...CWC....L..A.I.TK.R...VAQQ..M.Q...FS.H...FR.........I.H....VTVA**

**MmFADS4**  **.AQ..I.EIS...I.HH..S.W.VTI.ISF..TVS...CSF....L....M.KK......M.K..MC....LS.D...YR.....V...IYP.....DVG**

**ScaFADS1**  **.V.L....I.......Y....L....ISTA..AI....G..............K......V.K.I..............L....................**

**ScaFADS2**  **.G.LF..EFL.....SY...GWI.T..T.LIMATS...S.............RS........K.L...M...SGN....R.........I.N....V..L**

310 320 330 340 350 360 370 380 390 400

....|....|....|....|....|....|....|....|....|....|....|....|....|....|....|....|....|....|....|....|

**HsFADS1**  **PFFFALGKILSVELGKQKKKYMPYNHQHKYFFLIGPPALLPLYFQWYIFYFVIQRKKWVDLAWMITFYVRFFLTYVPLLGLKAFLGLFFIVRFLESNWFV**

**HsFADS2**  **H-V.V..EWQPI.Y..K.L..L......E........L.I.M...YQ.IMTM.VH.N......AVSY.I...I..I.FY.ILGA.LFLNFI.....H...**

**HsFADS3**  **.-V.L..ESS-..Y..K.RR.L...Q..L........L.TLVN.EVENLAYMLVCMQ.A..L.AAS..A....S.L.FY.VPGV.LF.VA..V...H...**

**MmFADS1**  **.L......V.P....RE...H...............................V...........LS..A.I.F..M......G.................**

**MmFADS2**  **H-V.V..EWQPL.Y..K.L..L......E........L.I.M...YQ.IMTM.S.RD......A.SY.M...Y..I.FY.ILGA.VFLNFI.....H...**

**MmFADS3**  **.-V.L..ESS-..Y..K.RR.L......L........L.TLVN.EVENLAYMLVCMQ.T..L.AAS..S....S.S.FY.ATGT.L..VA..V...H...**

**MmFADS4**  **.-L.LV.DTQPIKY..K.I..ID.EK..L..YMVAL.F.M.V..NLQSMQVMYL..Y.M.I..VSS..I.Y.I.FG.FY.IFGTVL.IYL.K.I..P.IA**

**ScaFADS1**  **.I..V...K....V..........QY.........................AV..RQ.......VS..I.IS...I....IQG..IML.L...V......**

**ScaFADS2**  **K-V.V..DVQP..F..R.I..L...Y..E........L.I.V..YIQ.MTTM.T.RD......AT.Y.I.Y.T....FF.FFGSIV.VVF...I..H...**

410 420 430 440 450 460 470 480 490 500

....|....|....|....|....|....|....|....|....|....|....|....|....|....|....|....|....|....|....|....|

**HsFADS1**  **WVTQMNHIPMHIDHDRNMDWVSTQLQATCNVHKSAFNDWFSGHLNFQIEHHLFPTMPRHNYHKVAPLVQSLCAKHGIEYQSKPLLSAFADIIHSLKESGQ**

**HsFADS2**  **........V.E..QEAYR..F.S..T.....EQ.F.........................L..I....K...........E....R.LL...R...K..K**

**HsFADS3**  **.I.......KE.G.EKHR....S..A.....EP.L.TN................R......SR.....K.......LS.EV..F.T.LV..VR...K..D**

**MmFADS1**  **.................V..............Q....N....................................Y..K.E.....T.....VY.......**

**MmFADS2**  **.......LV.E..L.HYR..F.S..A.....EQ.F.........................L..I....K...........E....R.LI..VS...K..E**

**MmFADS3**  **.I.......KE.G.EKHR..A.S..A.....EP.L.I........................RR.....KAF.....LH.EV..F.T.LV...G...K..D**

**MmFADS4**  **Y....S....KMSSEE.H..L...VV....IEQ.F.....T...........................K.......LQ.IN..I.K..G..VR...K.AS**

**ScaFADS1**  **..........M....QKE..LTM..H......Q.L.....T...................F.......R.......L.......FT.....V.......E**

**ScaFADS2**  **..........N..DEKHK..L.M..DS...IEQ.S..........................Y.I....K...D...LN..V....QG.K...G...K..D**

....|....

**HsFADS1**  **LWLDAYLHQ**

**HsFADS2**  **........K**

**HsFADS3**  **I........**

**MmFADS1**  **.........**

**MmFADS2**  **........K**

**MmFADS3**  **I........**

**MmFADS4**  **..MN..YE-**

**ScaFADS1**  **........K**

**ScaFADS2**  **........K**

**Supporting figure 1**
