# Supplementary material for: Functional Desaturase Fads1 (Δ5) and Fads2 (Δ6) Orthologues Evolved before the Origin of Jawed Vertebrates
Source: PLoS One. 2012 Feb 22;7(2):e31950. doi: 10.1371/journal.pone.0031950 (PMC3285190; doi:10.1371/journal.pone.0031950)
Supplement: Figure S2 — Fads2b and Fads2c intron-exon organization in Oryzias latipes . (DOCX) [file pone.0031950.s002.docx]

**Supporting figure 2**

[ENSORLP00000007987](http://www.ensembl.org/Oryzias_latipes/Transcript/ProteinSummary?db=core;g=ENSORLG00000006357;r=6:10925366-10927092;t=ENSORLT00000007988)

>Exon_Intron_Sequence

ATGGGAGGTGGAGGACAGCTGGCAGACACTCCGGAGGCTGGCAGCAGCAGAGATGCAGGTGTGTTTACCTGGGAGGAGGTGCAGAAGCACAGCAGCAGGAAGGATCAATGGCTGGTGATCAATCGAAAGGTTTATAACATCACTCAGTGGACCAAAAGACACCCAGGAGGGTCAAGTGTCATCAGCCTGTATGCTGGAGAAGATGCCACG

gtaaacatgaattcctgttaactttatgtggaaattattattattattattattattattannnnnnnnnnnnnnnnnnnnnnnnnnnnnnnnnnnnnnnnnnnnnnnnnnnnnnnnnnnnnnnnnnnnnnnnnnnnnnnnnnnnnnnnnnnnnnnnnnnnnnnnnnnnnnnnnnnnnnnnnnnnnnnnnnnnnnnnnnnnnnnnnnnnnnnnnnnnnnnnnnnnnnnnnnnnnnnnnnnnnnnnnnnnnnnnnnnnnnnnnnnnnnnnnnnatttattttagtaatattatcatttcggcctgaatagtttcctgtgacacaacgataaatatgaaataatgccttttaacacattatacacttccttcatttatttagtttatattaaaacaaatagttatattgaatcaatttctaaaataaagaacttcatttggataaatgcagtaattagaattgcatcacatgaagccttttgtaaacatttcaaactgatacgatatcaatatgtgatgtttaactcttgttctcagactgacatgagtgaaactagtaattaagtcattgtaccatgacagtgaattattggaaatgggtacaaattaaagaaattgtgaccactgtccacccacctactgtttcattccttagatgtcccccagttcttaaagtaaggcaaaatttggatttttgatactttctgcatcttatacatcatgtttgcgttttattttatttttttttattccagGAGGCCTTCACAGCTTTTCATCCTGATCTAAATTTTGTGCGAAAGTTTCTAAAGCCTCTTCTGATCGGGGAGCTGGCAGCAGCAGAGCCCAGCCAGGATCCAAACAAGAATgtgagctgtcagattatctttgattgtctttcgcctaaacaaagaggcccctcaataattaggctgcattgcttcattaagctttttcttcttccagATTGCAATCATAGAGGATTTTGAAAGATTACGAGTTCAGGCAGAGAAAGATGGTTTGTTTAAGACAAAGCCTTTGTTCTTCTGCCTCCATTTGGGTCACATCCTCCTGCTGGAGatcctcgcgtggacccttgtctggctctgggggacaggttggacagtgacattactgtgtgcggcattgctggcaacttcacaggtgaataaaacaaaagatcttttggttgagtaccttgcaatgaaatgttctaagtactttgattctgcaggcacaggctgggtggttgcagcatgactttggccacctttctgtctttaagaagtctaaatggaatcatcttgtgcacaagtttgtaattggacatttaaaggtaactttttagatcaattgaagtttttcctttatattttaaagtctccttacattttttttatgttttgtttttatttttagggagcctcagccagctggtggaatcatctacattaccgacatcattccaaacccaatgttctggataaagatcctgatgtcaatatgtcaggtctcttcgttttaggagccgttcagccagtggaa

gcaagtagctcagaatataaatacctccagtgcttcataaatgcatcgatcgaagctgctcaactaactctgttgatcttttcttcacagtacggcatcaaaaagatcaaacacatgccctacaatcaccaacaccagtacttttttctctgtatggatgcttaaagctgtttaaaaccccaaactgtcattgatttctccaagaatgaagtctgttttctgttccagtgggacctccattactcattccagtggtgttcaatcttcaaatactgacgaccatgatttcccgtcgtgattgggtggtaggatgtcaacctatgagacacaaagcgatttgatatgaaatccatatttgagctgaatttaaattaacccatctaaactgactaaaaaactatgaattatattttttttaaaaaccactcaatctgcttttttcaaaggatttggcttggtacgtgtcattctacctgcgctttttctactgttatatccccttttatggtttcctcggctcagtggcactcatcacctttgtcaggtaaagaagttgaatttttcttaaatttgatcaaagtaataaaaaaatttattaacatatttgttgtcccgtgctttaccttaatatacattttttccaggttcctggagagtcactggtttgtgtgggtgactcagatgaaccatttgccaatggagatcgatcacgagaggcgtcaggaatggctgaccacacaggtatgtttgtcattcaaagtctatgtatcctgacgcctacatactataagctcctactccatctgtctaggggcacatttttagggttttttgccctcaccgcttgatttttttagaaaatctcagcaggtattttgtgtaagattttcttgttgggttgtgttggccaccattcatgagcaaatatccataatgaatgacatacaacacttagatgacacttttatcagtagcaaaaggccgtaaaagtaaaaactctttttttacaaagagacaacattttgaagattaccaaaacaaaaaaaagttctgtcttgcatgaagtacacaatcttcagctcaaataccgttagaagcgttcaagtgtttgttttactatacaggctgaactgtgcttgtcattgtggttatttttttattcttcaaacatctgtttcatctaccaaagacaagagaaaagacgggagcagttcagcagagagaagactaaacaaaacacatcagaagccttgacagagagaggctccctgtcacagctttgaaaagacgttaagtctgttaaaaagaaaacaacaactttgagaatgtctgcagttactgttccatttccctaacatgcatttttagttggtttctttatttcatttgttcataaaagatagcgacaaacagaattgtacattttctaggatatattttgctcctgtcacttccacttaattaaataaataacaatatttatgtaaaatatgtacaaatgtaagagattaatttcagcaaaaatgagcaaggcagaatagaaatgattcaaagaatcaaaacctttacattcaaaagtttactagatgttaaactttgaaacgtcccattagactcttgtgcaaaagacaaccaagtaaatagactattagtttgtttacagttgtgttgtttttttcatacatgtattctgcagtattgcttcaaaaagaaggacttgaacagatgcattaactcattcatacctgggccttcatcttttttttctttttttagctccaggctacttgtaacattgaacagtccttcttcaacgactggttcagcggacatctcaacttccaaattgaacaccagtaagtttagtttttgagtcctccatacaaaccaggaaatacagtcaaactatcattttccatctccacacttatgtcaatgaaacgttttcaataaatgtatcttgttaaataaaattgataataagaaaaacagctttttgatggtgtatcttgatccatatgggagccaaatatttgttattagccccaatacaactgtgatacatagactagtaaattatgagaacattaaagagtccaataaagttctactttttgctggttttctgactcttcacattttgtttcagtttgtttcccagaatgcctcgtcacagttaccacctggtggctccacaggtgcgtgaactctgtaagaagcatggagttccttaccaaataaaatccttatggagggccatggctgatgttgtcaggtatgccatctccacaagaaacacataatgttaaaaatgaaataaaacattgaccatgtgactttgtgtctttaagcacttcagccagaatgtccgctgtctcccgttacaaacagatcaaatgcttaactgacaaagaggctgctattttattcatgacctgacctgtttatttatttttttctaaccttctttttcagctcgctgaagacctcaggggatctctggctggacgcatacctccataaatgacaaccttcatcttaaagaagtcctgctcgttttccattgtagcaaaattcagccaattaggttgtgtttttcttatgaatagtagacttactcaaacgcaataaagtgtgttctcatgtgacatgttgatcaactctgatgttttgaagagcaaatttactactactgatttgagacatccaagtaattatactgataagtaccataggatgacttattcaattaacttccaggatgtttttgtgttaaacctctaacttatgctacaattattgttttatgttctatctatactcaataaaaattgtgttgaccactccttgctttcttagcagcataaatcaacacctattcagataagaagtcataataaaggatgtccagaaaatttagagtggatattgcagatgatataatggtttaactcaaaaaaaggaggaagaaggaacttttgaatgcaacaattcttgttaatttgtgcatagatgaatctgactctgaaagttaacttttatttaactctgaacatttttatctaaaagcttgataaattaaaagcaataaagtctgactcatggcatgtgtgctacaaatgacttcatctctttgatcataaggtacagtgtggtgcaatgtaaatgaagtcagacagtatgaagtggaataagctccaaagtagcattacctgctgcaacttgacaaactacaagggtcagtcatttgagaagaaaacagtcaattcttttttatttttggtttgggtcgtctccacaaataaggttttacacatgtggttaaccatggtgctattttagatgaccccacccttacaataacgtgttctccctaccatgacaaaggtggataaaggtggaaatatttcatgtaatccatggacaccagtgaagatcacaaatcattgaagaaaaaaggttcagcgcactgtctagtgggtctagatgacccaactcccaacgttaaagtgcctaggatagcacaagggttaag

>Exon_Sequence

ATGGGAGGTGGAGGACAGCTGGCAGACACTCCGGAGGCTGGCAGCAGCAGAGATGCAGGTGTGTTTACCTGGGAGGAGGTGCAGAAGCACAGCAGCAGGAAGGATCAATGGCTGGTGATCAATCGAAAGGTTTATAACATCACTCAGTGGACCAAAAGACACCCAGGAGGGTCAAGTGTCATCAGCCTGTATGCTGGAGAAGATGCCACG

GAGGCCTTCACAGCTTTTCATCCTGATCTAAATTTTGTGCGAAAGTTTCTAAAGCCTCTTCTGATCGGGGAGCTGGCAGCAGCAGAGCCCAGCCAGGATCCAAACAAGAATATTGCAATCATAGAGGATTTTGAAAGATTACGAGTTCAGGCAGAGAAAGATGGTTTGTTTAAGACAAAGCCTTTGTTCTTCTGCCTCCATTTGGGTCACATCCTCCTGCTGGAGatcctcgcgtggacccttgtctggctctgggggacaggttggacagtgacattactgtgtgcggcattgctggcaacttcacaggcacaggctgggtggttgcagcatgactttggccacctttctgtctttaagaagtctaaatggaatcatcttgtgcacaagtttgtaattggacatttaaagggagcctcagccagctggtggaatcatctacattaccgacatcattccaaacccaatgttctggataaagatcctgatgtcaatatgtcaggtctcttcgttttaggagccgttcagccagtggaatacggcatcaaaaagatcaaacacatgccctacaatcaccaacaccagtacttttttctctgtggacctccattactcattccagtggtgttcaatcttcaaatactgacgaccatgatttcccgtcgtgattgggtggatttggcttggtacgtgtcattctacctgcgctttttctactgttatatccccttttatggtttcctcggctcagtggcactcatcacctttgtcaggttcctggagagtcactggtttgtgtgggtgactcagatgaaccatttgccaatggagatcgatcacgagaggcgtcaggaatggctgaccacacagctccaggctacttgtaacattgaacagtccttcttcaacgactggttcagcggacatctcaacttccaaattgaacaccagttgtttcccagaatgcctcgtcacagttaccacctggtggctccacaggtgcgtgaactctgtaagaagcatggagttccttaccaaataaaatccttatggagggccatggctgatgttgtcagctcgctgaagacctcaggggatctctggctggacgcatacctccataaatga

>Protein_Sequence

[M](http://web.expasy.org/cgi-bin/translate/dna_sequences?/work/expasy/tmp/http/seqdna.8839,1,1)GGGGQLADTPEAGSSRDAGVFTWEEVQKHSSRKDQWLVINRKVYNITQWTKRHPGGSSVISLYAGEDATEAFTAFHPDLNFVRKFLKPLLIGELAAAEPSQDPNKNIAIIEDFERLRVQAEKDGLFKTKPLFFCLHLGHILLLEILAWTLVWLWGTGWTVTLLCAALLATSQAQAGWLQHDFGHLSVFKKSKWNHLVHKFVIGHLKGASASWWNHLHYRHHSKPNVLDKDPDVN[M](http://web.expasy.org/cgi-bin/translate/dna_sequences?/work/expasy/tmp/http/seqdna.8839,1,236)SGLFVLGAVQPVEYGIKKIKH[M](http://web.expasy.org/cgi-bin/translate/dna_sequences?/work/expasy/tmp/http/seqdna.8839,1,258)PYNHQHQYFFLCGPPLLIPVVFNLQILTT[M](http://web.expasy.org/cgi-bin/translate/dna_sequences?/work/expasy/tmp/http/seqdna.8839,1,288)ISRRDWVDLAWYVSFYLRFFYCYIPFYGFLGSVALITFVRFLESHWFVWVTQ[M](http://web.expasy.org/cgi-bin/translate/dna_sequences?/work/expasy/tmp/http/seqdna.8839,1,341)NHLP[M](http://web.expasy.org/cgi-bin/translate/dna_sequences?/work/expasy/tmp/http/seqdna.8839,1,346)EIDHERRQEWLTTQLQATCNIEQSFFNDWFSGHLNFQIEHQLFPR[M](http://web.expasy.org/cgi-bin/translate/dna_sequences?/work/expasy/tmp/http/seqdna.8839,1,392)PRHSYHLVAPQVRELCKKHGVPYQIKSLWRA[M](http://web.expasy.org/cgi-bin/translate/dna_sequences?/work/expasy/tmp/http/seqdna.8839,1,424)ADVVSSLKTSGDLWLDAYLHK

[ENSORLP00000007988](http://www.ensembl.org/Oryzias_latipes/Transcript/ProteinSummary?db=core;g=ENSORLG00000006358;r=6:10932230-10934821;t=ENSORLT00000007989)

>Exon_Intron_Sequence

ATGGGAGGTGGAGGTCAGCAGACAGAGCCGGTCAGCGGCAAAGGGACTGGTTTTTTCACCTGGGAAGAGGTGCAGAAGCACAGCAACAGGAACGATCAGTGGCTGGTGGTCAAACGAAAAGTTTACAACGTCACAAATTGGGCCAAAAGACACCCAGGGGGGTTTCGAGTCATCAGCCACTATGCAGGAGAGGATGCCACGgtgagtatgtttcatcttaacatgcatgtcaggacaactgaatctttgctgcacatttcattgggacagtcagtgggcattttgtgcaggacttttggaaaactaagaggatcagcagttatactaatctggcatatttatactagtgagcaatcgctccacggaataaaacaatgtgtcaataattaataaaaaacaaattaaaaacactagtaatacatagaaacctgaaatctgtgttagctcttttggaaaatgcccttaaatgtatggcaagctgtttgaaccaaaaaaaaaaatagtagtcaaaaatggggaaaaactactaagtaaaatctttgtaatgagaatgaacatttcatatactggatcaccaagcaaactgcaaaaaattacgtaaactaaaagataaactgagtttccacaacttttttattttctgtagtcatgaaacgcatctgcacataaatccactctcccaacatttgtcaaataatgtttatccaataattattaaactagattttgtttttgatagaaatccattgcaactactgcaacatagcataaatcatatccatattaaaaactagattacacaaaaaaaacaacgtttttttggcactaagcaaatataattagttttaagcagtgcaaagtccaaagtttcctggtttgtcaccatcatcagttttctgtgccatgtttttctttttgtgtagtaggctaataaatgcaaatatccttgaaatgccaatgaggctgaccttgacctttgtacagggtttttacttgtaattgaacatgacctgctggctaactaagaacagggttctgttagcttgtcactgcaactgtttatgatatggatgctgaacttcctaaattgtttaggtttcaaactttacttctattctgagtaataaaatattgctaaggacaaatgctgttgttgtaaatgtactcatactggattgttaatgcacctgatccagttcaatggtaagttacgtatcagcatttgtagatactttgcacaatcccttaatttttgtcatttagtcagagaaaatacaattttgtaaatgaaaagtacacttaactaggctcagcaaattaaaatagggaactaaaatgcttttgcctttcagGAGGTGTTTGCTGCTTTTCATCCAGATCAGGACTTTGTTCAAAAGTTTCTAAAGCCTCTGCACATTGGAGAGTTGGCAGCAACAGAGCCCAGCCAGGACAGAGAAAAGAATgtgagttttttttttggattcaaggagttgttgaagctcttgtgtgattcacagatcttacataaaaagatttgtgtttccagcctttcaggactatcctttatgtcttgtgctttccagGATTCCATAATAAAAGATTTTGATTCCCTGCGAGTCCAGACAGAAAAAGAAGGTTTGTTTAAGGCTAAGCCTTTGTTTTTCCTCCTTCATCTGGGTCACATCATACTGCTGGAAGCACTCGCATGGCTCTCTGTTTGTTACTGGGGAACCAGCTGGACAATGACTTTCCTGTGTGCAACACTGCTGGCAACTGCTCAGgtagttttaaacacagttatctttttgatgatgaaaatatttccagttctaacagttatttatctttaaattctgcagGCACAGGCAGGGTGGTTGCAGCATGACTTTGGTCATCTATCTGTCTTTAGGAAGTCTAAATGGAATCACCTCGTACACAAATTTGTCATTGGACATTTAAAGgtaacttttctttagataatgtaggaatttaaccagaaaacagacatagatgtaaagatttttttctttgaaagGGAGCTTCAGCCAATTGGTGGAATCATAGACATTTCCAGCATCATGCCAAACCCAATATCTTTAGGAAGGACCCTGATATTAATGTGATGGACGTCTTTGTACTTGGaaacactcaaccagtagaggcaagtttctccaagtaaaatatttttttagtttactgagctttaaagcttactcacctctattttcttactacagtatggaataaaaaagatcaagaaaatgccctacaatcgccaacacgagtatttcttcctttgtatgtattcatagatgttaaaaatgttacaaatcatagcagtgtacttaaaaagaactattgtctttgcatttcttagtgggtccacctctactcattccagtattctacaattttaacatattttacaccatgattacccgtcgtgactgggtggtaagcctgtattttctttttggatcaatttggtttattataagaaatgtatgcattcatttaacacaactactgaaacctaacagttatgttactaaaataaacatttacatttttttaaggatctgtcttgggcaatgacgtattactttcgctactatttctgctttgtacctctgtatggcttctggggctcatttgcactccagttgtttgccaggtgagctaaaataatatagtttatttcttgttttttcttttgcttgtcattaatgatctgcttcctatcatcaaaattgacttttgacaattttaatacccagcacagagagaaatttaaagtgaagccctttgatttcccagatttttggagagtcactggtttgtgtgggtaactcaaatgaaccatttaccaatggagatggagcatgagaagcggcaggactggatgaccacacaggtaatgcatttctctaaaaacatagtttcaccattttataattctgtaccagagttcaaaaaaattatggttggaaagtgattaatcttggttctgccattgaaatcctgacatttgtttgatgtaattttcatgcagctatccgttcttgatgtctttatttctatcccgtaatcggatgaattcacaaggtctttgcttgtgctcacagtcaaaacataagacaaaatagtcgggagttttttattttttttaacccagattgcactgacacgtttggggactgtgtaaaaaggttggctgtacttcactgagtcccaactgttgtttcacagttgttctatacagttttatgtgtctaatattaaaataatattgtgtcctttggaaatcttatgactagagacaccttagcattggcaatgtattaatttttggttcgtaattggttggttggaggttcaaatcctaggctgttagtatgttcttgggtaattcaaaagtctccctcaaatgtgaaaaatatgtttctattcttaaaaccaacatgacaatgtgtagaaatatgaaagtaaatatttttaatattgacagattaaaatgcccaaaacagaagttcacttgagtttattctacacctgatagataaaagagagtagaatattactaaatgagcaatacttatctgttatttgtttcaatggcatacgacacgtttagtgccattttgaaaggagataaatgaaacttaagtgtaatttgaggtctgtttttctgagccaaatgctaaaaagtgacagctgtgtgatggaggatgaacacagctacatttacattttgtctcctttttcagctggtgtccacttgtaatgttgaacagtccttcttcaatgactggttctctggacacctcaactttcaaattgaacaccagtaagtaaagttacaattacaggacctttgtcatgaagtctgatttgattttctttaataactcttttttttccccccgtcaagaataccaaaaaacaaaagaaaatactgatgtcaggttaaacacttgttctgtttctttctttcaagcttgtttcccaggatgccgcgtcataactaccacttggtggcgccacaagtccgtgcactgtgtgagaaacatggaattccttatgagatgaaatccttatggagaggcatggtggatgttgtgaggtaaggacaaggaaaaacagtgtgtgaggttcatgatgctgcaaaatcaagtcattgtgctgatttggttaccagagcaacaaggggaagatgtttttagtgacaaaacaataattcttgtttgtttaaatgtatatcaaattaatttttatcttatccttttttttaggtctctgaaaaagtcaggggatctgtggcttgatgcatatcttcataaatgacaaaatgggcctggaaggaggtgttgcataaaattagatagcacattttagcctttgagaattcctgtattcatactagaatcctctttcctttttgcaggtgctgaaaaggattttctgttgatacataggtgttcatttaatgtaattgttagaggtctttttttgttgttgttgctaaatttttgaggagaggggatgatttcaaacaaattttaccttgaaacaattacagatacaagaagggagcattattat

>Exon_Sequence

ATGGGAGGTGGAGGTCAGCAGACAGAGCCGGTCAGCGGCAAAGGGACTGGTTTTTTCACCTGGGAAGAGGTGCAGAAGCACAGCAACAGGAACGATCAGTGGCTGGTGGTCAAACGAAAAGTTTACAACGTCACAAATTGGGCCAAAAGACACCCAGGGGGGTTTCGAGTCATCAGCCACTATGCAGGAGAGGATGCCACGGAGGTGTTTGCTGCTTTTCATCCAGATCAGGACTTTGTTCAAAAGTTTCTAAAGCCTCTGCACATTGGAGAGTTGGCAGCAACAGAGCCCAGCCAGGACAGAGAAAAGAATGATTCCATAATAAAAGATTTTGATTCCCTGCGAGTCCAGACAGAAAAAGAAGGTTTGTTTAAGGCTAAGCCTTTGTTTTTCCTCCTTCATCTGGGTCACATCATACTGCTGGAAGCACTCGCATGGCTCTCTGTTTGTTACTGGGGAACCAGCTGGACAATGACTTTCCTGTGTGCAACACTGCTGGCAACTGCTCAGGCACAGGCAGGGTGGTTGCAGCATGACTTTGGTCATCTATCTGTCTTTAGGAAGTCTAAATGGAATCACCTCGTACACAAATTTGTCATTGGACATTTAAAGGGAGCTTCAGCCAATTGGTGGAATCATAGACATTTCCAGCATCATGCCAAACCCAATATCTTTAGGAAGGACCCTGATATTAATGTGATGGACGTCTTTGTACTTGGaaacactcaaccagtagagTatggaataaaaaagatcaagaaaatgccctacaatcgccaacacgagtatttcttcctttgtGgtccacctctactcattccagtattctacaattttaacatattttacaccatgattacccgtcgtgactgggtgGatctgtcttgggcaatgacgtattactttcgctactatttctgctttgtacctctgtatggcttctggggctcatttgcactccagttgtttgccaggTttttggagagtcactggtttgtgtgggtaactcaaatgaaccatttaccaatggagatggagcatgagaagcggcaggactggatgaccacacagCtggtgtccacttgtaatgttgaacagtccttcttcaatgactggttctctggacacctcaactttcaaattgaacaccag**Ttgtttcccaggatgccgcgtcataactaccacttggtggcgccacaagtccgtgcactgtgtgagaaacatggaattccttatgagatgaaatccttatggagaggcatggtggatgttgtgagg**Tctctgaaaaagtcaggggatctgtggcttgatgcatatcttcataaatga

>Protein_Sequence

[M](http://web.expasy.org/cgi-bin/translate/dna_sequences?/work/expasy/tmp/http/seqdna.309,1,1)GGGGQQTEPVSGKGTGFFTWEEVQKHSNRNDQWLVVKRKVYNVTNWAKRHPGGFRVISHYAGEDATEVFAAFHPDQDFVQKFLKPLHIGELAATEPSQDREKNDSIIKDFDSLRVQTEKEGLFKAKPLFFLLHLGHIILLEALAWLSVCYWGTSWT[M](http://web.expasy.org/cgi-bin/translate/dna_sequences?/work/expasy/tmp/http/seqdna.309,1,158)TFLCATLLATAQAQAGWLQHDFGHLSVFRKSKWNHLVHKFVIGHLKGASANWWNHRHFQHHAKPNIFRKDPDINV[M](http://web.expasy.org/cgi-bin/translate/dna_sequences?/work/expasy/tmp/http/seqdna.309,1,234)DVFVLGNTQPVEYGIKKIKK[M](http://web.expasy.org/cgi-bin/translate/dna_sequences?/work/expasy/tmp/http/seqdna.309,1,255)PYNRQHEYFFLCGPPLLIPVFYNFNIFYT[M](http://web.expasy.org/cgi-bin/translate/dna_sequences?/work/expasy/tmp/http/seqdna.309,1,285)ITRRDWVDLSWA[M](http://web.expasy.org/cgi-bin/translate/dna_sequences?/work/expasy/tmp/http/seqdna.309,1,298)TYYFRYYFCFVPLYGFWGSFALQLFARFLESHWFVWVTQ[M](http://web.expasy.org/cgi-bin/translate/dna_sequences?/work/expasy/tmp/http/seqdna.309,1,338)NHLP[M](http://web.expasy.org/cgi-bin/translate/dna_sequences?/work/expasy/tmp/http/seqdna.309,1,343)E[M](http://web.expasy.org/cgi-bin/translate/dna_sequences?/work/expasy/tmp/http/seqdna.309,1,345)EHEKRQDW[M](http://web.expasy.org/cgi-bin/translate/dna_sequences?/work/expasy/tmp/http/seqdna.309,1,354)TTQLVSTCNVEQSFFNDWFSGHLNFQIEHQLFPR[M](http://web.expasy.org/cgi-bin/translate/dna_sequences?/work/expasy/tmp/http/seqdna.309,1,389)PRHNYHLVAPQVRALCEKHGIPYE[M](http://web.expasy.org/cgi-bin/translate/dna_sequences?/work/expasy/tmp/http/seqdna.309,1,414)KSLWRG[M](http://web.expasy.org/cgi-bin/translate/dna_sequences?/work/expasy/tmp/http/seqdna.309,1,421)VDVVRSLKKSGDLWLDAYLHK
