# Supplementary material for: Functional Desaturase Fads1 (Δ5) and Fads2 (Δ6) Orthologues Evolved before the Origin of Jawed Vertebrates
Source: PLoS One. 2012 Feb 22;7(2):e31950. doi: 10.1371/journal.pone.0031950 (PMC3285190; doi:10.1371/journal.pone.0031950)
Supplement: Table S1 — List of identified Fads sequences and the accession numbers for all the sequences used in the phylogenetic analysis. (DOC) [file pone.0031950.s003.doc]

| **Species** | **Ensembl Protein prediction/**  **Accession Number** | | **Gene Name** |
| --- | --- | --- | --- |
| ***Homo sapiens*** | [ENSP00000322229](http://www.ensembl.org/Homo_sapiens/Transcript/Sequence_Protein?db=core;g=ENSG00000149485;t=ENST00000350997) | | *Fads1* |
|  | [ENSP00000278840](http://www.ensembl.org/Homo_sapiens/Transcript/Sequence_Protein?db=core;g=ENSG00000134824;t=ENST00000278840) | | *Fads2* |
|  | [ENSP00000278829](http://www.ensembl.org/Homo_sapiens/Transcript/Sequence_Protein?db=core;g=ENSG00000221968;t=ENST00000278829) | | *Fads3* |
| ***Macaca mulatta*** | [ENSMMUP00000018145](http://www.ensembl.org/Macaca_mulatta/Transcript/Sequence_Protein?db=core;g=ENSMMUG00000013798;t=ENSMMUT00000019375) | | *Fads1* |
|  | [ENSMMUP00000000439](http://www.ensembl.org/Macaca_mulatta/Transcript/Sequence_Protein?db=core;g=ENSMMUG00000000327;t=ENSMMUT00000000475) | | *Fads2* |
|  | [ENSMMUP00000000438](http://www.ensembl.org/Macaca_mulatta/Transcript/Sequence_Protein?db=core;g=ENSMMUG00000000328;t=ENSMMUT00000000474) | | *Fads3* |
|  | [ENSMMUP00000006822](http://www.ensembl.org/Macaca_mulatta/Transcript/Sequence_Protein?db=core;g=ENSMMUG00000005151;t=ENSMMUT00000007259) | | *Fads4* |
| ***Oryctolagus cuniculus*** | [ENSOCUP00000002863](http://www.ensembl.org/Oryctolagus_cuniculus/Transcript/Sequence_Protein?db=core;g=ENSOCUG00000003292;t=ENSOCUT00000003298) | | *Fads1* |
|  | [ENSOCUP00000003036](http://www.ensembl.org/Oryctolagus_cuniculus/Transcript/Sequence_Protein?db=core;g=ENSOCUG00000003499;t=ENSOCUT00000003499) | | *Fads2* |
|  | [ENSOCUP00000020609](http://www.ensembl.org/Oryctolagus_cuniculus/Transcript/Sequence_Protein?db=core;g=ENSOCUG00000014364;t=ENSOCUT00000029225) | | *Fads3* |
|  | [ENSOCUP00000016774](http://www.ensembl.org/Oryctolagus_cuniculus/Transcript/Sequence_Protein?db=core;g=ENSOCUG00000027838;t=ENSOCUT00000029573) | | *Fads4* |
| ***Canis lupus familiaris*** | [ENSCAFP00000023539](http://www.ensembl.org/Canis_familiaris/Transcript/Sequence_Protein?db=core;g=ENSCAFG00000025083;t=ENSCAFT00000025353) | | *Fads1* |
|  | [ENSCAFP00000023629](http://www.ensembl.org/Canis_familiaris/Transcript/Sequence_Protein?db=core;g=ENSCAFG00000016019;t=ENSCAFT00000025448) | | *Fads2* |
|  | [ENSCAFP00000023511](http://www.ensembl.org/Canis_familiaris/Transcript/Sequence_Protein?db=core;g=ENSCAFG00000015963;t=ENSCAFT00000025326) | | *Fads3* |
| ***Mus musculus*** | [ENSMUSP00000010807](http://www.ensembl.org/Mus_musculus/Transcript/Sequence_Protein?db=core;g=ENSMUSG00000010663;t=ENSMUST00000010807) | | *Fads1* |
|  | [ENSMUSP00000025567](http://www.ensembl.org/Mus_musculus/Transcript/Sequence_Protein?db=core;g=ENSMUSG00000024665;t=ENSMUST00000025567) | | *Fads2* |
|  | [ENSMUSP00000111659](http://www.ensembl.org/Mus_musculus/Transcript/Sequence_Protein?db=core;g=ENSMUSG00000024664;t=ENSMUST00000115995) | | *Fads3* |
|  | [ENSMUSP00000097507](http://www.ensembl.org/Mus_musculus/Transcript/Sequence_Protein?db=core;g=ENSMUSG00000075217;t=ENSMUST00000099923) | | *Fads4* |
| ***Monodelphis domestica*** | [ENSMODP00000004589](http://www.ensembl.org/Monodelphis_domestica/Transcript/Sequence_Protein?db=core;g=ENSMODG00000018682;t=ENSMODT00000004689) | | *Fads1* |
|  | [ENSMODP00000009301](http://www.ensembl.org/Monodelphis_domestica/Transcript/Sequence_Protein?db=core;g=ENSMODG00000007494;t=ENSMODT00000009482) | | *Fads2* |
|  | [ENSMODP00000009324](http://www.ensembl.org/Monodelphis_domestica/Transcript/Sequence_Protein?db=core;g=ENSMODG00000007513;t=ENSMODT00000009506) | | *Fads3* |
| ***Ornithorhynchus anatinus*** | [ENSOANP00000012512](http://www.ensembl.org/Ornithorhynchus_anatinus/Transcript/Sequence_Protein?db=core;g=ENSOANG00000007855;t=ENSOANT00000012514) | | *Fads1* |
|  | [ENSOANP00000020344](http://www.ensembl.org/Ornithorhynchus_anatinus/Transcript/Sequence_Protein?db=core;g=ENSOANG00000012862;t=ENSOANT00000020347) | | *Fads2* |
|  | [ENSOANP00000006525](http://www.ensembl.org/Ornithorhynchus_anatinus/Transcript/Sequence_Protein?db=core;g=ENSOANG00000004111;t=ENSOANT00000006527) | | *Fads3* |
|  | [ENSOANP00000012687](http://www.ensembl.org/Ornithorhynchus_anatinus/Transcript/Sequence_Protein?db=core;g=ENSOANG00000007978;t=ENSOANT00000012689) | | *Fads4* |
| ***Anolis carolinensis*** | XP_003224167 | | *Fads1a* |
|  | XP_003224189 | | *Fads1b* |
|  | XP_003224188 | | *Fads1c* |
|  | XP_003224187 | | *Fads1d* |
|  | XP_003224186 | | *Fads1e* |
|  | XP_003224168 | | *Fads2* |
| ***Gallus gallus*** | XP_421052 | | *Fads1a* |
|  | XP_426408 | | *Fads1b* |
|  | XP_421051 | | *Fads1c* |
|  | NP_001153900 | | *Fads2* |
| ***Xenopus tropicalis*** | [ENSXETP00000061474](http://www.ensembl.org/Xenopus_tropicalis/Transcript/Sequence_Protein?db=core;g=ENSXETG00000030682;t=ENSXETT00000064056) | | *Fads1* |
|  | [ENSXETP00000061225](http://www.ensembl.org/Xenopus_tropicalis/Transcript/Sequence_Protein?db=core;g=ENSXETG00000009043;t=ENSXETT00000065908) | | *Fads2* |
|  | [ENSXETP00000061790](http://www.ensembl.org/Xenopus_tropicalis/Transcript/Sequence_Protein?db=core;g=ENSXETG00000033437;t=ENSXETT00000064232) | | *Fads-like* |
| ***Oryzias latipes*** | [ENSORLP00000007982](http://www.ensembl.org/Oryzias_latipes/Transcript/Sequence_Protein?db=core;g=ENSORLG00000006353;t=ENSORLT00000007983) | | *Fads2a* |
|  | [ENSORLP00000007987](http://www.ensembl.org/Oryzias_latipes/Transcript/Sequence_Protein?db=core;g=ENSORLG00000006357;t=ENSORLT00000007988) | | *Fads2b* |
|  | [ENSORLP00000007988](http://www.ensembl.org/Oryzias_latipes/Transcript/Sequence_Protein?db=core;g=ENSORLG00000006358;t=ENSORLT00000007989) | | *Fads2c* |
| ***Gasterosteus aculeatus*** | [ENSGACP00000007209](http://www.ensembl.org/Gasterosteus_aculeatus/Transcript/ProteinSummary?db=core;g=ENSGACG00000005442;r=groupXIX:6935626-6936815;t=ENSGACT00000007227) | | *Fads2* |
| ***Danio rerio*** | [ENSDARP00000022396](http://www.ensembl.org/Danio_rerio/Transcript/Sequence_Protein?db=core;g=ENSDARG00000019532;t=ENSDART00000023278) | | *Fads2* |
| ***Rachycentron canadum*** | ACJ65149 | | *Fads2* |
| ***Lates calcarifer*** | ACS91458 | | *Fads2* |
| ***Dicentrarchus labrax*** | ACD10793 | | *Fads2* |
| ***Sparus aurata*** | AAL17639 | | *Fads2* |
| ***Siganus canaliculatus*** | ADJ29913 | | *Fads24* |
|  | ABR12315 | | *Fads26* |
| ***Scophthalmus maximus*** | AAS49163 | | *Fads2* |
| ***Anguilla japonica*** | ACI32415 | | *Fads2* |
| ***Salmo salar*** | NP_001117014 | | *Fads2* |
|  | NP_001165251 | | *Fads2a* |
|  | NP_001165752 | | *Fads2b* |
|  | NP_001117047 | | *Fads2c* |
| ***Scyliorhinus canicula*** | JN657543 | | *Fads1* |
|  | JN657544 | | *Fads2* |
| ***Branchiostoma floridae*** | | XP_002586930 | *Fads-like* |
