# Supplementary material for: Functional Desaturase Fads1 (Δ5) and Fads2 (Δ6) Orthologues Evolved before the Origin of Jawed Vertebrates
Source: PLoS One. 2012 Feb 22;7(2):e31950. doi: 10.1371/journal.pone.0031950 (PMC3285190; doi:10.1371/journal.pone.0031950)
Supplement: Table S2 — List of primers used to isolate and characterize Fads1 and Fads2 genes in Scyliorhinus canicula . (DOC) [file pone.0031950.s004.doc]

| **Designation** | **Sequence** | **Use** |
| --- | --- | --- |
| FADS1F1 | 5’ CCGTGTTCTCCACATCCaartggaayca 3’ | Gene isolation |
| FADS1F2 | 5’ CAAGGACCCCGACATCaayatgcaycc 3’ |
| FADS1R1 | 5’ TTGGTCTGGTACTCGATGccrtgyttngc 3’ |
| FADS1RaceF | 5’ TCATCTGAGTCACCCACACAAACCA 3’ |
| FADS1RaceR | 5’ CACTGCTCCCCCTCTACTTCCAATG 3’ |
| FADS1F2race | 5’ CGTTGAGGTGGGGAAACAGAAGAAGA 3’ |
| FADS1R2race | 5’ GAAAGACACCATCCAGGCCAAGTCC 3’ |
| FADS1RaceF3 | 5’ GTTTCCCACGATGCCAAGGCATAAC 3’ |
| FADS1RaceR1 | 5’ GGTGCCGGTTTACTTTTCCAAGATTCC 3’ |
| FADS2F1Sc | 5’GAGGACCGACAGGtggytngtnat 3’ |
| FADS2R1Sc | 5’ TGGCCGGAGAACcartcrttraa 3’ |
| FADS2F2Sc | 5’ GCGCCTCCGCCAAytggtggaayc 3’ |
| FADS2R2Sc | 5’ CTGGAAGTGCCGGtgrttccacca 3’ |
| FADS2RaceR | 5’ AGATAAGGTCTGGGTGGAAAGCCTGGA 3’ |
| FADS2RaceF | 5’ CCGCTACTTCACCACCTACGTCCCTTT 3’ |
| ScaFADS1Fcons | 5’ CCCAAGCTTAGGATGggctcgagtgcggag 3’ | Functional assay |
| ScaFADS1Rcons | 5’ CCGGAATTCtcacttatgtagatatgc 3’ |
| ScaFADS2Fcons | 5’ CCCAAGCTTAGGATGgggaaaggaggagag 3’ |
| ScaFADS2Rcons | 5’ CCGGAATTCtcatttgtggaggtatgc 3’ |
